# Supplementary material for: Specific cancer-associated mutations in the switch III region of Ras increase tumorigenicity by nanocluster augmentation
Source: eLife. 2015 Aug 14;4:e08905. doi: 10.7554/eLife.08905 (PMC4563131; doi:10.7554/eLife.08905)
Supplement: Supplementary file 1. — Thermodynamic and kinetic parameters of H-ras mutant in vitro experiments. Table contains background corrected GEF-dependent Eu3+-GTP association (kon), and dissociation (koff) kinetics of wt H-ras and mutants. The Kon, Koff, and Kd derived from Koff/Kon values are calculated from corrected association and dissociation data as described in ‘Materials and methods’. Dissociation constants (Kd) between mantGTPγS and wt H-ras and its mutants (in the presence of EDTA and in the absence of Mg2+). The absence of Mg2+ increases the Kd, which is otherwise in the pM range (John et al., 1988, 1990). Dissociation constants (Kd) of C-Raf-RBD and mantGTPγS-bound wt H-ras and its mutants were measured by fluorescence anisotropy as described in ‘Materials and methods’. Raw data are shown in Figure 2—figure supplement 2. DOI: http://dx.doi.org/10.7554/eLife.08905.017 [file elife08905s001.docx]

| H-ras | Background corrected Kon (M^-1^ s^-1^)  H-ras/ Eu^3+^-GTP (10 nM)  (+GEF) | Background corrected Koff (s^-1^)  H-ras/ Eu^3+^-GTP (10 nM)  +100μM GTP  (+GEF) | Kd (nM) derived from Koff/Kon  H-ras/ Eu^3+^-GTP  (+GEF) | Kd (nM)  mantGTPγS (100 nM) / H-ras  (+EDTA, -Mg^2+^) | Kd (nM)  H-ras mantGTPγS(100 nM) / RBD |
| --- | --- | --- | --- | --- | --- |
| wt | 3.76±0.11 *10^5^ | 0.00342±0.00009 | 9.1±0.4 | 207±19 | 417±52 |
| D47A/E49A | 4.29±0.17 *10^5^ | 0.00140±0.00011 | 3.3±0.3 | 854±110 | 636±98 |
| G48R | 3.76±0.11 *10^5^ | 0.00310±0.00005 | 8.2±0.3 | 251±19 | 534±74 |
| G48R/D92N | 3.36±0.08 *10^5^ | 0.00224±0.00004 | 6.7±0.2 | 439±46 | 410±80 |

| H-ras | Kon  (normalized to wt)  H-ras/ Eu^3+^-GTP (10 nM)  (+GEF) | Koff  (normalized to wt)  H-ras/ Eu^3+^-GTP (10 nM)  (+GEF) | Kd derived from Koff/Kon  (normalized to wt)H-ras/ Eu^3+^-GTP  (+GEF) | Kd  (normalized to wt)  mantGTPγS (100 nM) /H-ras  (+EDTA, -Mg^2+^) | Kd  (normalized to wt)  H-ras mantGTPγS (100 nM)/ RBD |
| --- | --- | --- | --- | --- | --- |
| wt | 1.00±0.04 | 1.00±0.04 | 1.00±0.05 | 1.00±0.13 | 1.00±0.18 |
| D47A/E49A | 1.14±0.06 | 0.41±0.04 | 0.36±0.04 | 4.13±0.65 | 1.53±0.30 |
| G48R | 1.00±0.04 | 0.91±0.03 | 0.91±0.05 | 1.21±0.14 | 1.28±0.24 |
| G48R/D92N | 0.89±0.03 | 0.65±0.03 | 0.73±0.04 | 2.12±0.30 | 0.98±0.23 |

**Supplementary file 1. Thermodynamic and kinetic parameters of H-ras mutant *in vitro* experiments**
